# Radiocapitellar incongruity of the radial head in magnetic resonance imaging correlates with pathologic changes of the lateral elbow stabilizers in lateral epicondylitis

**DOI:** 10.1371/journal.pone.0254037

**Published:** 2021-07-07

**Authors:** Yeun Soo Kim, Sung Taeck Kim, Kyoung Hwan Lee, Joong Mo Ahn, Hyun Sik Gong

**Affiliations:** 1 Department of Orthopedic Surgery, National Police Hospital, Seoul, Republic of Korea; 2 Department of Orthopedic Surgery, Seoul National University Bundang Hospital, Seoul National University College of Medicine, Seongnam, Republic of Korea; 3 Department of Radiology, Seoul National University Bundang Hospital, Seoul National University College of Medicine, Seongnam, Republic of Korea; Paracelsus Medizinische Privatuniversitat - Nurnberg, GERMANY

## Abstract

**Objective:**

Post-traumatic posterolateral rotatory instability (PLRI) can be shown as radiocapitellar incongruity or posterior translation (PT) of the radial head in magnetic resonance imaging (MRI). We aimed to evaluate whether PT correlated with pathologic changes of lateral elbow stabilizers in patients with lateral epicondylitis.

**Materials and methods:**

In MRIs of 160 patients with lateral epicondylitis, we measured PT of the radial head in the sagittal images. We qualitatively graded five lesions of the lateral elbow structures that included common extensor tendon (CET) lesion (grade 1–3), lateral collateral ligament complex (LCLC) insufficiency (grade 0–2), and absence or presence of bone marrow signal change, osteochondral lesion, and calcification. We analyzed whether the PT correlated with pathologic changes of the lateral elbow stabilizers and evaluated the diagnostic value of the PT for severe lesions.

**Results:**

The average PT was 1.9 mm. The PT correlated with both the CET lesion (*p* < 0.001) and LCLC insufficiency (*p* < 0.001). The optimal cutoff values of the PT for grade 3 CET lesion and grade 2 LCLC lesion were 2.6 and 2.8 mm, respectively. When potential PLRI was defined as the PT of > 3.4mm as suggested for post-traumatic PLRI, 21 patients had potential PLRI. The positive predictive values of the PT > 3.4mm were 76% for grade 3 CET lesions and 67% for grade 2 LCLC insufficiency.

**Conclusion:**

This study demonstrates that PT of the radial head correlates with pathological changes of the lateral elbow stabilizers. As radiocapitellar incongruity is easy to measure quantitatively, it can be used for screening potential PLRI in patients with lateral epicondylitis.

## Introduction

Lateral epicondylitis, recently called lateral epicondylopathy, is known to be caused by chronic overuse of the lateral forearm muscles, especially the extensor carpi radialis brevis, which arises from the lateral epicondyle of the humerus [1]. Posterolateral rotatory instability (PLRI) of the elbow usually occurs as a result of trauma, in which the ulna and radius rotate posterolaterally against the humerus. This kind of instability can also be present in patients with lateral epicondylitis, resulting from a degenerative change of the lateral stabilizers as well as from iatrogenic injury from steroid injection or surgical debridement [2–5]. However, the diagnosis of PLRI is not easy without procedures such as arthroscopic examination or examination under anesthesia [6–8].

Although the diagnosis of lateral epicondylitis is usually made from physical examination and taking a history, magnetic resonance imaging (MRI) can be helpful for evaluating other causes of pain and the extent of the lesion for surgical treatment. MRI findings of lateral epicondylitis include degenerative changes or tears of the common extensor tendon (CET) origin and lateral collateral ligament complex (LCLC) [9, 10].

For post-traumatic PLRI, Hackl et al. quantitatively measured radiocapitellar incongruity or posterior translation (PT) of the radial head in sagittal MRI images of the elbow [11]. They suggested that PT greater than 2mm is highly suspicious of elbow instability and if it is more than 3.4 mm, a reliable confirmation of diagnosis could be obtained for PLRI [11]. We hypothesized that radiocapitellar incongruity would be also helpful for assessing elbow instability in patients presenting with lateral epicondylitis. Therefore, the purpose of this study was to investigate whether PT of the radial head correlated with pathological changes of the lateral elbow stabilizers in patients with lateral epicondylitis, and to assess its diagnostic value for assessing severe lesions.

## Materials and methods

### Patient selection

This study was approved by Seoul National University Bundang Hospital Institutional Review Board (B-1912/582-104). Informed consent was waived by the institutional review board (the data were analyzed anonymously). We reviewed 178 consecutive patients who underwent MRI for further evaluation of lateral epicondylitis in a training referral hospital between September 2016 and August 2019. All patients presented with chronic lateral elbow pain that had lasted for more than 6 months. At the initial visit, we evaluate clinical features including symptom duration, history of trauma, and generalized ligament laxity. The clinical evaluation was performed by a single surgeon (HSG) with 20 years of experience, one of the authors. We excluded patients who had a history of trauma within the past 6 months from the time of MRI scans and generalized ligament laxity with a Beighton score of 4 or higher. All patients underwent standardized antero-posterior and lateral X-rays. Patients with a carrying angle of fewer than 5 degrees or greater than 25 degrees and those with osteoarthritis of Broberg-Morrey grade 2 or higher were excluded. Patients who were unable to maintain their elbows in a fully extended and supinated position during the MRI examination were also excluded from the study.

### Assessment of imaging parameters

In plain radiographs, we evaluated for angular deformity, sequelae of old trauma, and calcification. A 3.0-T system (Ingenia or Ingenia Cx, Philips Healthcare, the Netherlands) with a dedicated elbow-coil was used to perform the MRI. Axial and coronal T1-weighted (TR/TE, 550–750/8–15) and fat-suppressed T2-weighted fast spin-echo sequences (2,900–3,200/75–100) were performed at 2.5-mm slice thickness, 0.25mm intersection gap, 120×120 mm field of view, and 256×256 imaging matrix in all subjects. Patients were asked to fully extend the elbow with forearm supination during MRI. We first confirmed whether the coronal image was constructed parallel to the trans-condylar axis and the elbow was in full extension and supination. We measured the angle between the longitudinal axis of the distal humerus and the proximal ulna in the sagittal MR image. The cut-off angle for full extension was 10 degrees of flexion contracture. Since there has been no detailed discussion on how to measure the rotation of the forearm in the elbow MR image, we estimated the forearm rotation through the direction of the radial tuberosity. The posterior margin of the radial tuberosity is known to be directed an average of 16.6 degrees forward in a fully supinated position [12]. By confirming this on sagittal MR images, Patients with forearm pronation more than 15 degrees were excluded from the study.

**Fig 1:**
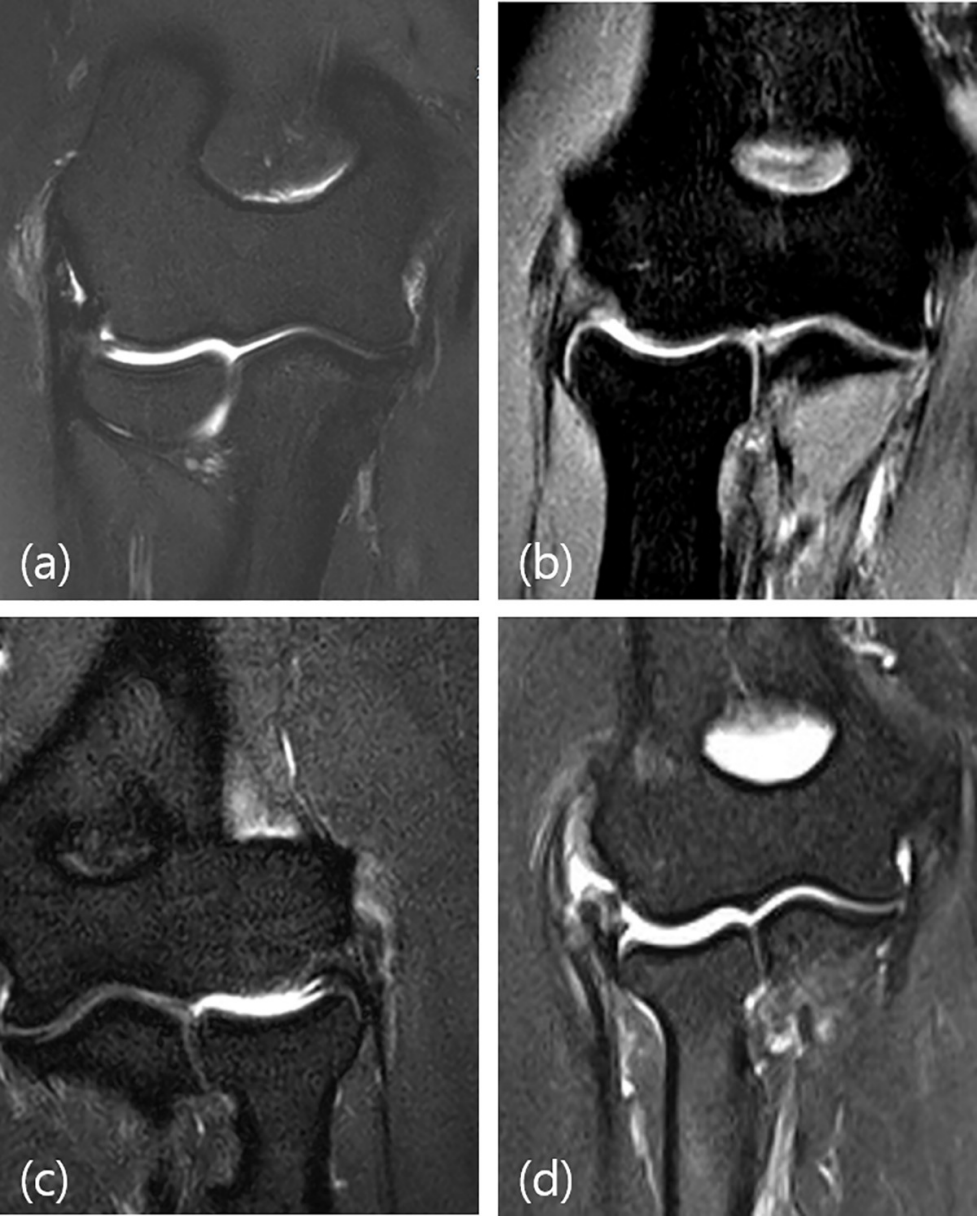
a. 41-year-old woman with tendinosis of the common extensor tendon (CET). b. 59-year-old man with intermediate-grade partial tear of the CET and partial tear of the lateral collateral ligament complex (LCLC). c. 48-year-old woman with high-grade partial tear of the CET and partial tear of the LCLC. d. 54-year-old woman with near-complete tear of the CET and LCLC.

**Fig 2:**
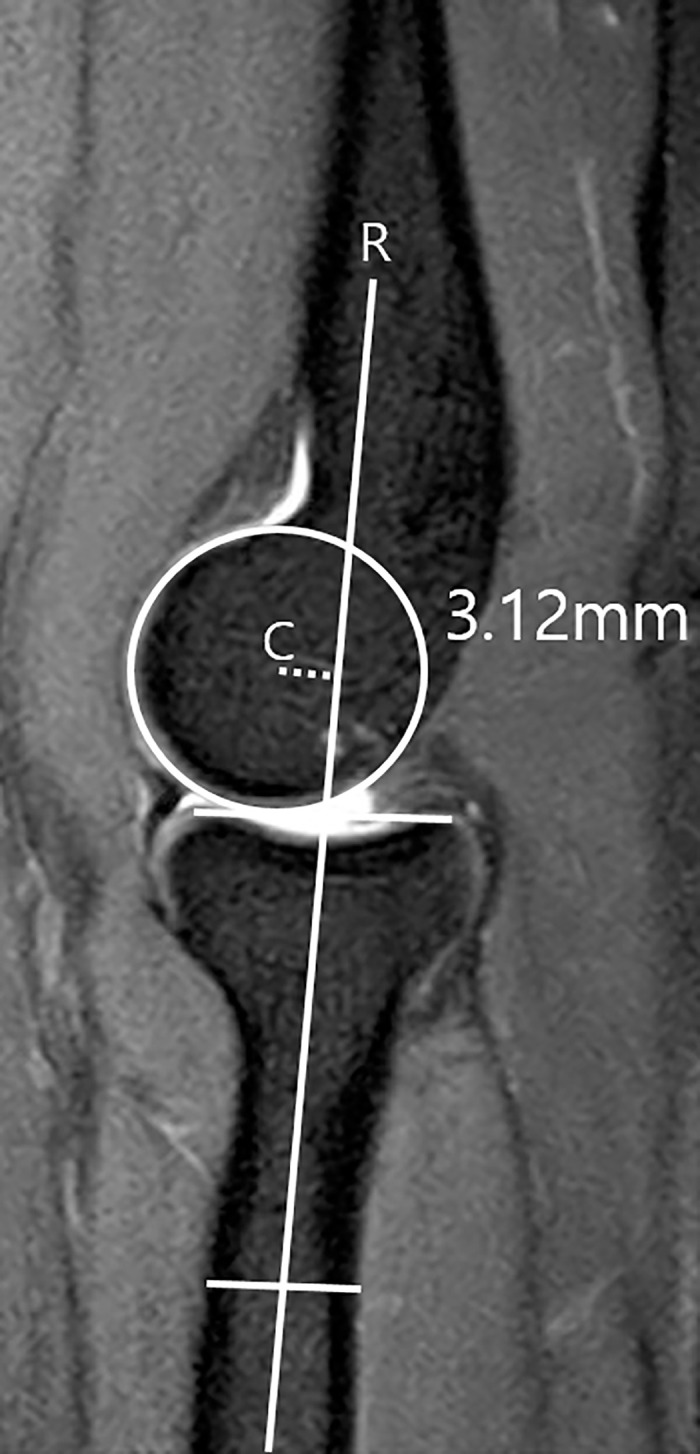
Measurement of radiocapitellar incongruity of the radial head. In sagittal image through the center of the radial head, the center of the capitellum (C) and the longitudinal axis of the proximal radius (R) were marked. The minimum distance between (C) and (R) was measured.

The CET lesion and ligamentous abnormality were evaluated in T2 weighted coronal and axial images. Proton density sequences were excluded in evaluating these structures, to avoid the magic angle phenomenon. Following previous studies, we defined tendinosis as thickening of the tendon or increase in the signal intensity of the tendon not higher than the fluid signal. Partial thickness tears (PTTs) were categorized as low-, intermediate- and high-grade tears. We defined low-grade PTT as tearing less than 20% of tendon thickness, intermediate-grade PTT as 20 to 80%, and high-grade PTT as more than 80%. A full-thickness tear (FTT) was defined as a gap of the fluid signal-intensity across the tendon substance [9, 10, 13]. CET lesions were rated as 1 = tendinosis or low-grade PTT, 2 = intermediate-grade PTT, 3 = high-grade PTT or FTT (Fig 1A–1D). The LCLC was evaluated as 0 = normal, 1 = partial tear, thickening or thinning of the ligament, and 2 = near complete or complete tear (Fig 1A–1D). Osteochondral lesions and bone marrow signal changes were identified in proton density weighted images and fat suppressed T2 weighted fast spin echo images, respectively. Abnormality was defined as presence of an osteochondral lesion or increase of bone-marrow signal around the radio-capitellar joint. Calcification was defined as the presence of calcium deposits in the CET substance. PT of the radial head was measured in a T2 or proton density weighted sagittal image. We measured the minimum distance between the longitudinal axis of the proximal radius through the center of the radial head and the center of the capitellum (Fig 2).

The reading protocol was developed by two musculoskeletal radiologists, who had 8 (YK) and 28 (JMA) years of experience, based on previous literatures; each protocol was then taught to two orthopedic surgeons with 8 (YSK) and 4 (STK) years of experience in reading elbow MR images. These two surgeons read all images in a randomized order after being blinded to any clinical information including age, sex, course of the disease, and past medical history. Then one of the observers re-evaluated all randomly ordered radiologic parameters two months later for intra-observer reliability. The kappa values of 0.41–0.60 could be accepted as fair, 0.61–0.80 as good, 0.81–1.00 as excellent agreement in the reliability test. As a result, the kappa values were 0.655 to 0.899 for inter-observer reliability and 0.797 to 0.911 for intra-observer reliability. Therefore, inter- and intra-observer reliabilities were considered to be good to excellent (Table 1).

**Table 1:** Cohen’s kappa of inter- and intra-observer reliability.

Variables	Inter-observer	Intra-observer
CET lesion	0.815	0.906
LCL complex lesion	0.899	0.791
Osteochondral lesion	0.877	0.791
BM signal change	0.655	0.910
Calcification	0.731	0.815
Posterior translation	0.847	0.911

CET, Common Extensor Tendon; LCLC, Lateral Collateral Ligament Complex; BM, Bone Marrow.

### Statistical analysis

In univariate analysis, we correlate the severity of the lesion of the lateral elbow stabilizers and other radiologic parameters including osteochondral lesion, bone marrow signal change, calcification, and posterior translation of the radial head. We performed the Jonckheere-Terpstra test for the comparison of continuous variables. Categorical variables were analyzed through the linear by linear association. Multinomial logistic regression analysis was performed to analyze factors that might be associated with the severity of CET and LCLC lesions respectively. We also calculated the optimal cutoff values of the PT for the diagnosis of grade 3 CET lesion and grade 2 LCLC insufficiency using receiver operating characteristic curve analysis.

In addition, we evaluated whether the PT of > 3.4mm, which was suggested for a reliable confirmation for post-traumatic PLRI, could be applied for lateral epicondylitis [11]. We assessed positive predictive values and negative predictive values of the PT > 3.4 mm for grade 3 CET lesion and grade 2 LCLC insufficiency, respectively.

Statistical correlations were set at *p* < 0.05, and all statistical analyzes were done using SPSS version 23 (IBM Corporation, Armonk, NY, USA) and R version 4.0.3 (The R foundation for statistical computing)

## Results

Among 178 patients who had undergone elbow MRI for clinically diagnosed lateral epicondylitis, 18 patients were excluded. Through history taking and physical examination, 2 patients with trauma history and 1 patient with generalized ligament laxity were excluded. In the review of X-ray findings, 5 patients with advanced osteoarthritis and 1 patient with cubitus varus were excluded. 9 patients were excluded due to the failure to maintain a fully extended and supinated position during MRI scans. Finally, 160 patients (64 men and 96 women) were analyzed for the study. The mean age was 53.6 years (range, 20 to 79; standard deviation, 9.6 years).

### Imaging parameters

A CET lesion was observed in all 160 patients to some degree. An LCLC lesion was present in 88 (55%) of the patients. Osteochondral lesion and bone-marrow signal change were detected in 7 and 15 elbows, respectively. Calcification was identified in 21 patients (Table 2). The incidence of LCLC lesions increased with the severity of CET lesions: An LCLC was preserved in 73% (37/51) of grade 1, 40% (31/78) of grade 2, and 13% (4/31) of grade 3 CET lesions. The average PT of the radial head was 1.9 mm (range, -0.6 ~ 5.4 mm; standard deviation, 1.1 mm).

**Table 2:** Distribution of radiologic findings.

Findings	Severity	*N* = 160
CET lesion	Grade 1	51
	Grade 2	78
	Grade 3	31
LCL complex lesion	Normal	72
	Grade 1	64
	Grade 2	24
Osteochondral lesion		7
BM signal change		15
Calcification		21

CET, Common Extensor Tendon; LCLC, Lateral Collateral Ligament Complex; BM, Bone Marrow.

### Parameters associated with the severity of CET and LCLC lesions

The degree of PT tended to increase with the severity of CET lesion (*p* < 0.001); the average PT was 1.4 mm in patients with a grade 1 CET lesion, 1.8 mm in grade 2, and 3.3 mm in grade 3. The degree of translation also increased with the severity of LCLC insufficiency (*p* < 0.001); The average PT was 1.3 mm in patients with normal LCLC, 2.1 mm in grade 1, and 3.3 mm in grade 2 (Table 3). The incidence of osteochondral lesion (p = 0.027) and bone-marrow signal change (p = 0.014) increased with the LCLC grade, not with CET grade. The presence of calcification was not correlated with both CET and LCLC grades (Table 3).

**Table 3:** Trend analysis for the severity of CET and LCLC lesions.

	CET lesion			LCLC lesion		
Variables	Severity	Mean±SD	*P-value*	Severity	Mean±SD	*P-value*
Posterior translation	Gr 1	1.39±0.76	< 0.001**	Normal	1.33±0.65	< 0.001**
	Gr 2	1.76±0.95		Gr 1	2.10±1.02	
	Gr 3	3.29±0.99		Gr 2	3.32±1.18	
		N (%)			N (%)	
Osteochondral lesion	Gr 1	2/51 (3.9%)	0.632	Normal	1/72 (1.4%)	0.027*
	Gr 2	3/78 (3.8%)		Gr 1	3/64 (4.7%)	
	Gr 3	2/31 (6.5%)		Gr 2	3/24 (12.5%)	
BM signal change	Gr 1	6/51 (11.8%)	0.737	Normal	4/72 (5.5%)	0.014*
	Gr 2	4/78 (5.1%)		Gr 1	5/64 (7.8%)	
	Gr 3	5/31 (16.1%)		Gr 2	6/24 (25%)	
Calcification	Gr 1	5/51 (9.8%)	0.126	Normal	10/72 (13.9%)	0.16
	Gr 2	9/78 (11.5%)		Gr 1	3/64 (4.7%)	
	Gr 3	7/24 (29.2%)		Gr 2	8/24 (33.3%)	

CET, Common Extensor Tendon; LCLC, Lateral Collateral Ligament Complex; BM, Bone Marrow.

Multinomial logistic regression indicated that the severity of CET and LCLC lesions was associated with the degree of the PT. Grade 2 LCLC lesion was also associated with bone-marrow signal change (Tables 4 and 5).

**Table 4:** Factors associated with the severity of the common extensor tendon lesion.

Variables	CET lesion	*p-value*	*OR*	*95% CI*
Age	Grade 2	0.241	1.025	0.984 ~ 1.067
	Grade 3	0.014*	1.092	1.018 ~ 1.171
Posterior translation	Grade 2	0.013*	1.805	1.134 ~ 2.875
	Grade 3	<0.001**	7.576	3.763 ~ 15.254

CET, Common Extensor Tendon; OR, Odd radio; CI, Confidence Interval.

**Table 5:** Factors associated with the severity of the lateral collateral ligamentous complex lesion.

Variables	LCLC lesion	*p-value*	*OR*	*95% CI*
Age	Grade 1	0.358	1.02	0.978 ~ 1.063
	Grade 2	0.025*	1.093	1.011 ~ 1.181
Posterior translation	Grade 1	<0.001*	3.127	1.865 ~ 5.242
	Grade 2	<0.001*	11.61	5.076 ~ 26.557
Bone marrow signal change	Grade 1	0.555	1.58	0.346 ~ 7.213
	Grade 2	0.026*	10.939	1.338 ~ 89.434

LCLC, Lateral Collateral Ligamentous Complex; OR, Odd radio; CI, Confidence Interval.

**Fig 3:**
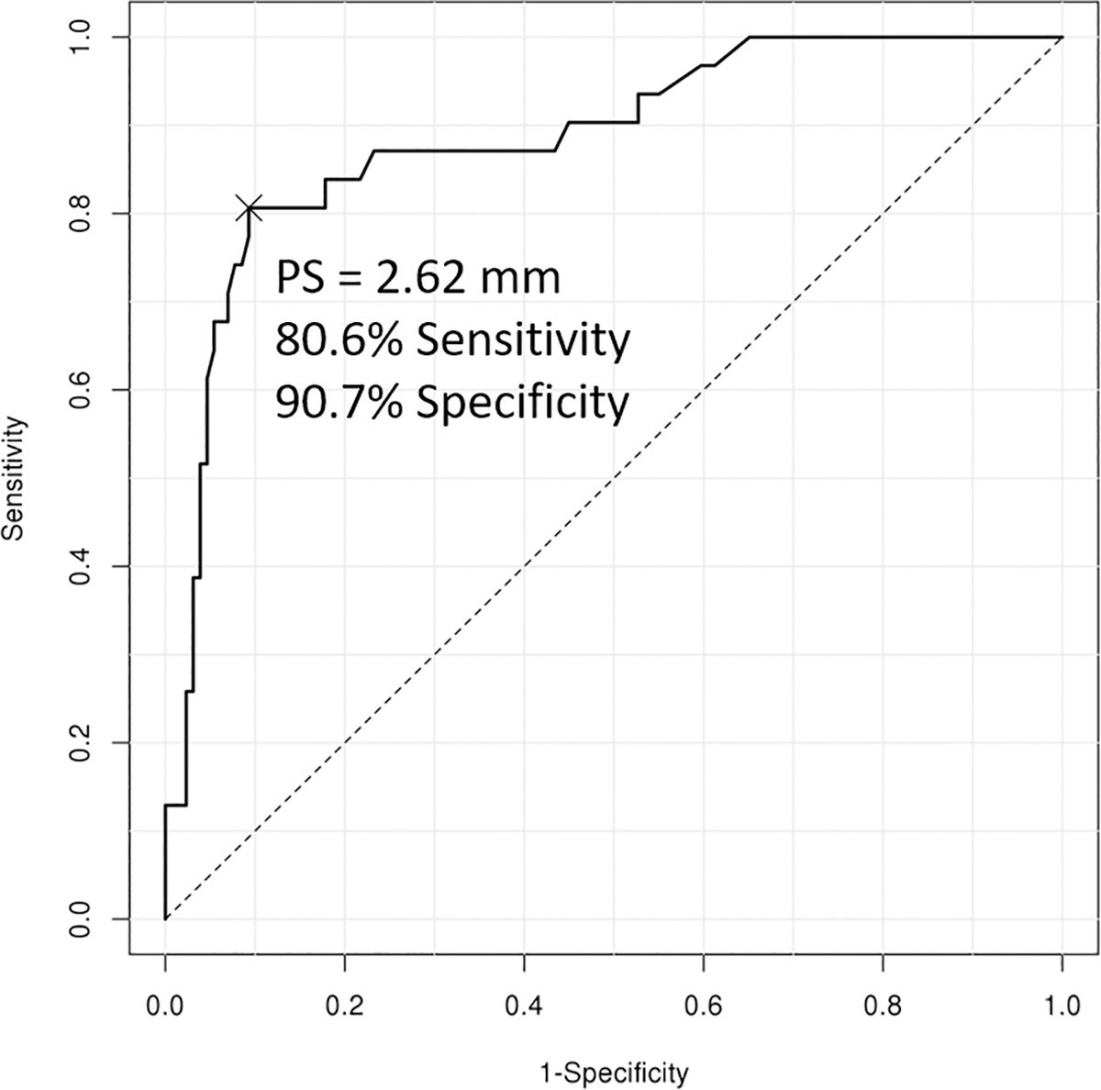
Receiver operating characteristic curve analysis of posterior translation of the radial head for grade 3 common extensor tendon lesion.

**Fig 4:**
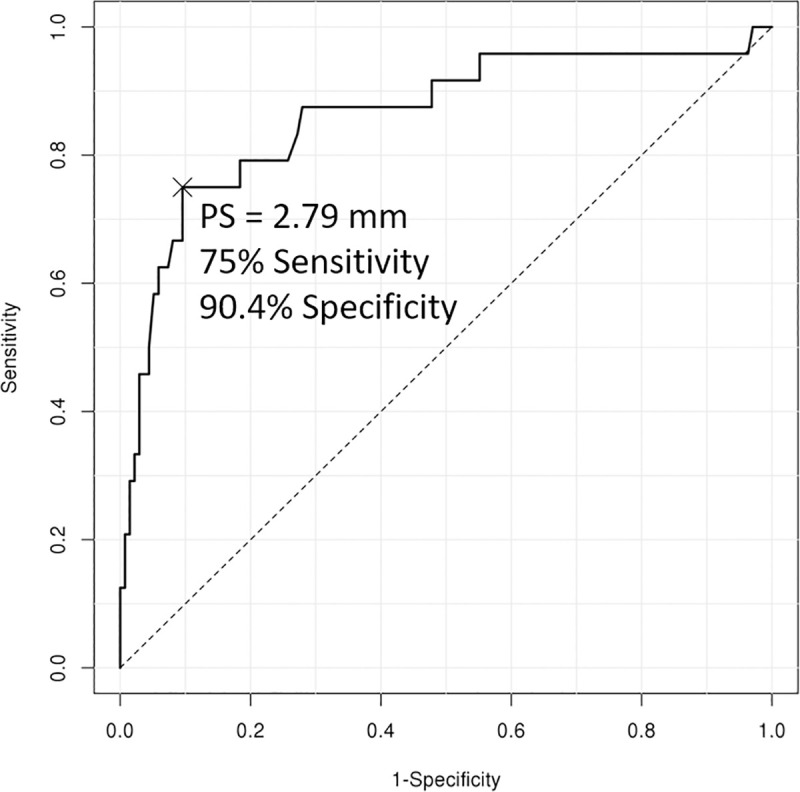
Receiver operating characteristic curve analysis of posterior translation of the radial head for grade 2 lateral collateral ligamentous complex lesion.

The optimal cutoff values of the PT for grade 3 CET lesion and grade 2 LCLC insufficiency were 2.6mm (sensitivity, 81%; specificity, 91%; AUC, 0.886; *p* < 0.001), and 2.8 mm (sensitivity, 75%; specificity, 90%; AUC, 0.861; *p* < 0.001), respectively (Figs 3 and 4).

### Potential posterolateral rotatory instability

**Fig 5:**
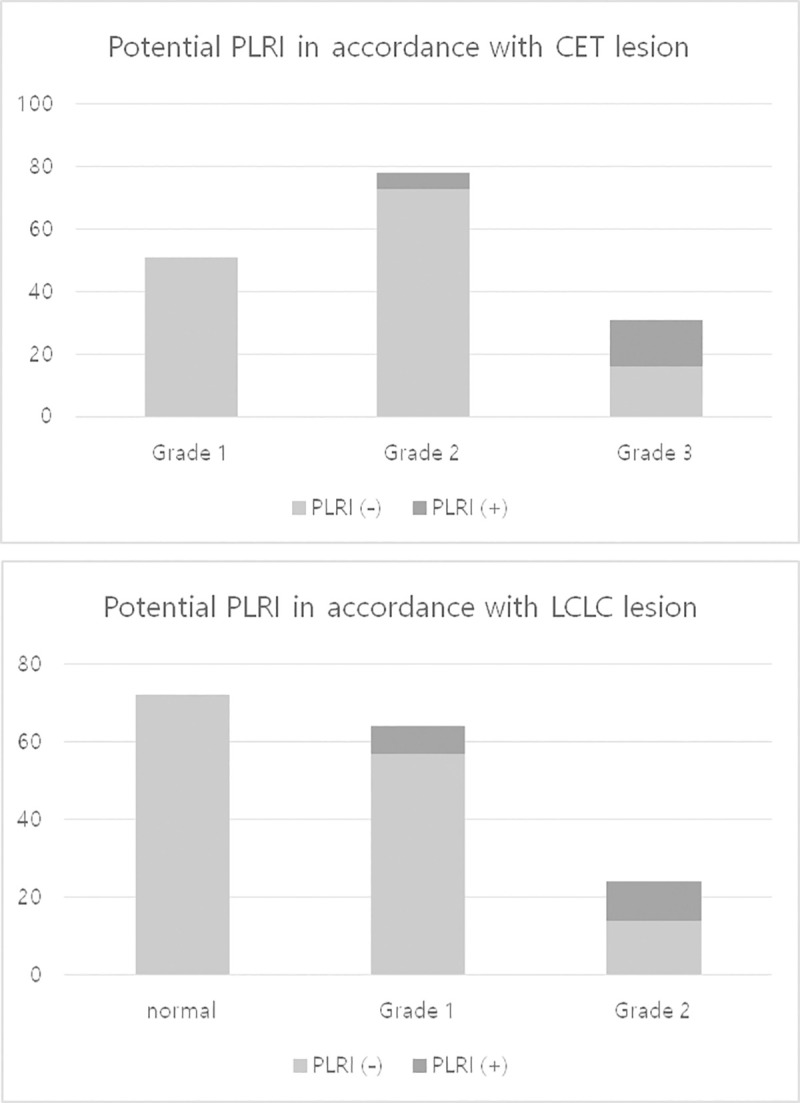
The incidence of potential posterolateral rotatory instability in accordance with the severity of the magnetic resonance imaging parameters. PLRI, Posterolateral Rotatory Instability; CET, Common Extensor Tendon; LCLC, Lateral Collateral Ligament Complex.

Potential PLRI was present in 21 patients (13%), as defined by the PT of > 3.4mm as a cut-off value. There was no patient with potential PLRI who had a grade 1 CET lesion or normal LCLC (Fig 5). Five out of 78 patients (6%) with grade 2 CET lesions and 16 out of 31 patients (52%) with grade 3 CET lesions had potential PLRI. Seven out of 64 patients (11%) with grade 1 LCLC insufficiency and 14 out of 24 patients (58%) with grade 2 LCLC insufficiency had potential PLRI.

The positive predictive values of the PT > 3.4mm were 76% for grade 3 CET lesions (Confidence interval (CI), 0.55 ~ 0.9) and 67% for grade 2 LCLC insufficiency (CI, 0.46 ~ 0.82). The negative predictive values were 89% for grade 3 CET lesions (CI, 0.86 ~ 0.91) and 93% for grade 2 LCLC insufficiency (CI, 0.9 ~ 0.95).

## Discussion

In this study, we evaluated whether radiocapitellar incongruity in sagittal MRI images correlates with pathological changes of the lateral elbow stabilizers. This study shows that the degree of PT of the radial head is independently associated with the severity of both LCLC and CET lesions.

We found that a CET lesion was present in all patients, and an LCLC lesion was observed in 55% of the patients, which is consistent with previous studies using MRI. According to a systematic review, an MR signal change of the CET was observed in 90% of lateral epicondylitis [14]. Bredella et al. reported that lateral ulnar collateral ligament abnormality in MRI was observed in 19 (54%) of 35 lateral epicondylitis patients, and all patients with severe lesions (complete tear of extensor carpi radialis brevis) had abnormalities of the ligament [9]. In our study, the incidence of LCLC lesions also increased with the severity of the CET lesions.

The diagnosis of PLRI has usually been made by arthroscopic examination or examination under anesthesia [6, 7]. Kwak et al. conducted examination under anesthesia on patients who had undergone surgical treatment for lateral epicondylitis [3]. They reported that a subtle instability caused by chronic LCLC injury was noted in the elbows with lateral epicondylitis, and preoperative MRI can be used to exclude the instability with its high negative predictive value, as is consistent with our study’s finding that no patient with potential PLRI had normal LCLC. They also addressed that the pre-operative visual analogue scale score was significantly higher in patient with instability [3]. In their study, a subtle instability was present in 17 (14%) of 122 patients, which is similar to the incidence in our study (13%).

To date, there have been few studies on radiologic evaluation for screening PLRI of the elbow. Hackl et al. compared MRI images of post-traumatic PLRI patients diagnosed with physical examination or arthroscopy with the control group, and suggested objective radiologic criteria for PLRI using radiocapitellar incongruity. They reported that if radiocapitellar incongruity > 2mm, PLRI could be highly suspected, and if > 3.4 mm, it could be confirmed [11]. We measured PT of the radial head in patients with lateral epicondylitis using the same method, and confirmed that radiocapitellar incongruity can be used as a meaningful indicator to assess the pathological changes of the lateral elbow stabilizers in lateral epicondylitis. As the footprint of the CET is oblique to the longitudinal axis of the distal humerus, it is difficult to quantitatively measure the extent of the lesion in MRI [15, 16]. Therefore, we consider that PT of the radial head, which can be quantitatively measured in MRI, can be useful for assessing the pathological changes of the lateral elbow stabilizers and for screening potential PLRI in lateral epicondylitis.

Both CET and LCLC are known as dynamic or static stabilizers involved in PLRI, but there is controversy over whether they should be the concern of treatment in lateral epicondylitis [5, 17, 18]. Kalainov et al. reported three cases of lateral epicondylitis with recurrent pain and symptomatic elbow instability. After debridement of the CET and reconstruction of the lateral elbow stabilizers, they reported that the pain and instability were resolved. They proposed corticosteroid injection and failed surgery as possible causes, suggesting that PLRI of the elbow should be considered when treating recalcitrant lateral epicondylitis [2]. Shim et al. reported good results with open debridement and ligament reconstruction in chronic lateral epicondylitis with apparent instability. They pointed out multiple steroid injections as a major risk factor [5]. In addition, Arrigoni et al. demonstrated relationship between ligamentous patholaxity and intra-articular abnormality, including synovitis and lateral capitellar chondropathy in lateral epicondylitis [19]. In our study, osteochondral lesions and bone-marrow signal changes were observed more frequently as the LCLC grade increased. On the other hand, van Kollenberg et al. compared 24 patients of lateral epicondylitis with the control group and reported no significant difference in signal changes of the lateral collateral ligament. They emphasized not to overestimate the abnormalities of the ligament and to avoid unnecessary surgical treatment [18].

There are several limitations to this study. First, this was a cross-sectional study and did not involve follow-up evaluations for the clinical implications of the MRI parameters. A longitudinal study would be necessary to find out their clinical significance. Second, we lacked confirmatory diagnostic tests for PLRI, such as arthroscopic examination or examination under anesthesia. Further studies are necessary to confirm the usefulness of this quantitative MRI assessment as well as the best cut-off values for diagnosing PLRI in patients with lateral epicondylitis.

## Conclusions

This study demonstrates that PT of the radial head in sagittal MRI images correlates with pathological changes of the lateral elbow stabilizers. As radiocapitellar incongruity is easy to measure quantitatively, it can be used for screening potential PLRI in patients with lateral epicondylitis.
